# Dietary Supplementation of Yeast Culture Into Pelleted Total Mixed Rations Improves the Growth Performance of Fattening Lambs

**DOI:** 10.3389/fvets.2021.657816

**Published:** 2021-05-12

**Authors:** Baijun Song, Tingting Wu, Peihua You, Hongze Wang, Jennifer L. Burke, Kun Kang, Wei Yu, Mengzhi Wang, Bo Li, Yuhua He, Qin Huo, Changsheng Li, Wannian Tian, Rongquan Li, Jianping Li, Chunqing Wang, Xuezhao Sun

**Affiliations:** ^1^The Innovation Centre of Ruminant Precision Nutrition and Smart and Ecological Farming, Jilin Agricultural Science and Technology University, Jilin City, China; ^2^Jilin Inter-regional Cooperation Centre for the Scientific and Technological Innovation of Ruminant Precision Nutrition and Smart and Ecological Farming, Jilin City, China; ^3^Portal Agri-Industries Co., Ltd., Nanjing, China; ^4^Angel Yeast Co., Ltd., Yichang, China; ^5^School of Agriculture and Environment, Massey University, Palmerston North, New Zealand; ^6^College of Animal Science and Technology, Yangzhou University, Yangzhou, China

**Keywords:** animal performance, carcass, digestibility, fattening lamb, high grain diet, pelleted total mixed ration, rumen fermentation parameters, yeast culture

## Abstract

There is a growing interest in the use of yeast (*Saccharomyces cerevisiae*) culture (YC) for the enhancement of growth performance and general animal health. Grain-based pelleted total mixed rations (TMR) are emerging in intensive sheep farming systems, but it is uncertain if the process of pelleting results in YC becoming ineffective. This study aimed to examine the effects of YC supplemented to pelleted TMR at two proportions of corn in the diet on animal performance, feed digestion, blood parameters, rumen fermentation, and microbial community in fattening lambs. A 2 × 2 factorial design was adopted with two experimental factors and two levels in each factor, resulting in four treatments: (1) low proportion of corn in the diet (LC; 350 g corn/kg diet) without YC, (2) LC with YC (5 g/kg diet), (3) high proportion of corn in the diet (HC; 600 g corn/kg diet) without YC, and (4) HC with YC. Fifty-six 3-month-old male F2 hybrids of thin-tailed sheep and Northeast fine-wool sheep with a liveweight of 19.9 ± 2.7 kg were randomly assigned to the four treatment groups with an equal number of animals in each group. The results showed that live yeast cells could not survive during pelleting, and thus, any biological effects of the YC were the result of feeding dead yeast and the metabolites of yeast fermentation rather than live yeast cells. The supplementation of YC resulted in 31.1 g/day more average daily gain regardless of the proportion of corn in the diet with unchanged feed intake during the 56-day growth measurement period. The digestibility of neutral detergent fibre and acid detergent fibre was increased, but the digestibility of dry matter, organic matter, and crude protein was not affected by YC. The supplementation of YC altered the rumen bacterial population and species, but the most abundant phyla *Bacteroidetes, Firmicutes*, and *Proteobacteria* remained unchanged. This study indicates that YC products can be supplemented to pelleted TMR for improved lamb growth performance, although live yeast cells are inactive after pelleting. The improved performance could be attributed to improved fibre digestibility.

## Introduction

Sheep is of economic importance in Northeast China (1). Sheep farming in the area is shifting from grazing or grazing plus supplements to intensive indoor feeding with a high proportion of grain included in the diet. The indoor feeding system enables feed additives to be extensively used for the manipulation of rumen fermentation and improved animal performance. There has been increasing pressure from both consumers and governments to remove antibiotics and synthetic chemical compounds from animal industries. This has led to increased research in the use of other feed additives as alternative growth promoters in sheep (2).

One of the common feed additives used by farmers and researchers is yeast culture (YC) (3), which is a natural product produced from yeast fermentation. YC contains, in addition to live and dead yeast cells, culture media and the products of metabolism, including proteins, polysaccharides (mainly β-glucans and mannan-oligosaccharides), lipids, vitamins, peptides, amino acids, nucleotides, organic acids, and antioxidants (4). YC supplementation has a range of beneficial effects, including increased feed intake (5), stabilisation of rumen pH (6, 7), increased feed digestion (8), enhancement of overall immunity (9, 10), and consequently improved animal performance (3, 5).

To increase growth rates in sheep production, farmers tend to increase the proportion of cereal grains in the diet (11). However, a high-grain diet can affect the rumen environment by reducing rumen pH, consequently resulting in disorders of the digestive system in ruminants (12, 13). Thus, one of the key goals when feeding sheep is to maintain the optimal rumen environment for growth. High-yielding dairy cows fed a large amount of grain in their diets can suffer the same problem. To solve the problem, YC is supplemented to the diet of dairy cows because of its role in the stabilisation of rumen pH (6, 7). YC may have a similar effect on the rumen pH of sheep when fed high-grain diets (14).

Feeding total mixed rations (TMR) has been popular due to its advantages of providing balanced nutrients in the diet, the relatively stable rumen environment, reduced feed sorting, and better animal performance in dairy cows (15). TMR feeding has been further developed by pelleting the TMR and fed to cattle (16–18) and sheep (19–23). Feeding of a pelleted TMR results in almost no feed selection by animals, non-palatable ingredients can be included in the diet, minimisation of feed loss during storage and feeding, and improved ease of feeding management and labour efficiency (11).

Pelleting is a critical step in the manufacture of pelleted TMR and involves steam conditioning, high temperature, and high pressure. These processes could cause changes to the nutritional value of the diet through chemical alterations (24) and could potentially eliminate the beneficial effects of YC. However, pelleting does not result in the disappearance of the beneficial effects of YC in non-ruminant animals (25, 26). Therefore, whether or not the effects of YC remain after pelleting when supplemented to TMR is uncertain.

We hypothesise that YC supplemented to pelleted TMR still has beneficial effects on fattening lambs. The objective of the study was to examine the effects of supplementing YC to pelleted TMR containing two proportions of corn in the diet on animal performance, digestibility, blood metabolites, rumen fermentation parameters, and ruminal microbial communities of fattening lambs.

## Materials and Methods

### Experimental Design and Animals

The experiment was conducted in a 2 × 2 factorial design with four treatments in total. The four treatments were (1) low proportion of corn in the diet (LC; 350 g corn/kg diet) without yeast (*Saccharomyces cerevisiae*) culture (YC) supplemented (CON), (2) LC with YC supplemented at the 5 g/kg diet, (3) high proportion of corn in the diet (HC; 600 g corn/kg diet) without YC, and (4) HC with YC supplemented at the 5 g/kg diet. The dose of YC at the 5 g/kg diet was recommended by the YC producer. The proportion of corn at the 350 g/kg diet was recommended by researchers (23), and the high proportion (600 g corn/kg diet) is used by farmers in the region.

Sixty male F2 hybrid lambs of thin-tailed sheep and Northeast fine-wool sheep, the most popular breed in the region (1), aged 3 months with a liveweight of 19.9 ± 2.7 kg, were used in the experiment. They were de-wormed, examined twice for brucellosis using the Rose Bengal plate test (*Bruceila* spp.) (27), and then adapted gradually from hay to pelleted TMR for 1 week and fed completely with pelleted TMR for another 2 weeks. All lambs were brucellosis negative. After the adaption period, the well-adapted and healthy lambs were randomly allocated to one of the four treatments after stratification for liveweight (*n* = 14 per treatment).

All lambs were individually weighed fortnightly for 8 weeks. The weighing was conducted at the same time of the day before the morning feeding each time using an electronic scale for livestock with a precision of 0.05 kg (Dahe Electronics Co., Ltd., Wuyi, Zhejiang, China). Average daily gain (ADG) was estimated using a plot of liveweight against time.

On day 56 after weighing, blood samples were collected randomly from six lambs from each treatment before the morning feeding. On day 70, rumen samples were randomly collected before the morning feed from 12 lambs to characterise the rumen microbial profile. On the same day, six lambs were randomly removed from each treatment for a digestibility trial and another six lambs were removed for slaughter. At the end of the digestibility trial, rumen samples were taken on day 82 at 3 h after morning feeding on these lambs for the determination of fermentation characteristics. Slaughter was performed on days 71 and 72 with three lambs each day for each treatment group. The remaining two lambs were released from the experiment on day 70.

### Feed and Feeding

The ingredients and nutrient contents of the experimental diets are presented in Table 1. According to the Chinese Feeding Standard of Meat-producing Sheep and Goats (NY/T 816-2004) (28), the diets were formulated with nitrogen content adjusted to be similar for the LC and HC diets. YC (®Bailike) specified for ruminants contained 200 g/kg mannan, 200 g/kg β-glucan, 200 g/kg crude protein (CP), and 80 g/kg moisture and was obtained from Angel Yeast Co., Ltd., Hubei, China. Premix, mineral supplements, and YC product were not ground, but other ingredients were ground to pass a 4/6 mm screen (a half part of the screen with holes having a diameter of 4 mm and another half with holes with a diameter of 6 mm). All ingredients were mixed thoroughly and steam conditioned for 10 s first, and then pelleting was performed at 85°C using a pelleting press (model YPM508E, Jiangsu Yongli Machinery Co., Ltd., Liyang, Jiangsu, China). Pelleted TMR was 3.2 mm in diameter. All the diets used in the experiment were produced at the Shanxi Subsidiary Company of Jiangsu Portal Agri-Industries Co., Ltd., China, in a single batch for each diet. The experimental diets were bagged and transferred to the experimental site and stored in a well-ventilated room for a month before use. At the beginning of the formal experimental period, feed samples were collected in triplicate for the count of live yeast.

**Table 1:** Ingredients and nutrient contents of experimental diets containing a low (LC) or high (HC) proportion of corn and supplemented with nil (CON) or yeast culture (YC).

**Corn**	**LC**		**HC**	
**Additive**	**CON**	**YC**	**CON**	**YC**
**Ingredient (kg/t of fresh weight)**				
Corn	350	350	600	600
Premix^a^ (Trace mineral salt and vitamins)	20	20	20	20
Corn germ meal	150	150	142	142
Sunflower seed meal	150	150	150	150
Millet husks	102	102		
Peanut shells	60	60		
Peanut vine	50	50		
Cottonseed meal	40	40	30	30
Barley malt rootlets	30	30	30	30
Bentonite	20	20		
Limestone	15	15	15	15
Calcium hydrogen phosphate	8	8	8	8
Sodium chloride	5	5	5	5
Yeast culture product (YC)		5		5
**Nutrient contents (g/kg of DM)**				
Dry matter (DM) (g/kg of fresh weight)	942	941	942	936
Organic matter (OM)	908	920	939	937
Crude protein (CP)	166	178	165	167
Neutral detergent fibre (NDF)	349	371	234	221
Acid detergent fibre (ADF)	207	205	90	86
Ether extract (EE)	28	28	35	35
Ca	12.2	12.0	9.9	11.0
P	5.6	5.6	5.5	5.8
Live yeast count (cfu/g) × 10^4^	<1	<1	<1	<1

*^a^Premix per kg contained 200,000 IU vitamin A, 60,000 IU vitamin D3, 550 mg vitamin E, 800 mg nicotinamide, 650 mg Cu (as CuSO_4_), 2,800 mg Fe (as FeSO_4_), 900 mg Mn (as MnSO_4_), 16 mg Se (as Na_2_SeO_3_), 3,600 mg Zn (as ZnSO_4_), 20 mg Co (as CoCl_2_), 15 mg [as Ca(IO_3_)_2_], and 15 g lysine. The carrier was composed of glucose, rice bran, zeolite powder, and limestone powder*.

All lambs were kept in the same room with a ventilation fan that operated for 24 h a day. No bedding was provided, but the pens were cleaned every morning before feeding. Lambs were offered feed twice a day with equal proportions at 08:00 and 16:00 h. Feed allowance was such that 10–20% of the feed offered was not eaten. Feed offered and refused was quantified daily for the estimation of feed intake. Lambs were group fed with 14 in each pen, except during the digestibility measurement period when lambs were individually fed in metabolic crates. Water was available at all times.

### Apparent Total Tract Digestibility Measurement

The digestibility trial began on day 70 and was carried out over 12 days. Lambs were given 6 days for adaption and 6 days for sample collection. Feed offered and refused, and the outputs of faeces and urine were collected and quantified daily using the total faeces collection technique (29). One-fifth of faeces was subsampled each day with half acidified for CP analysis only and another half not acidified for the analysis of other nutrients. The subsamples were pooled for each animal over the duration of the 6-day sampling period. Urine was collected in a bucket containing 100 ml of 6 M H_2_SO_4_ and stirred twice daily to homogenise urine and acid. Urine volume was measured, and one-tenth was subsampled and pooled for each animal. Faeces and urine subsamples were stored at −20°C. Feed refusals were not subsampled daily but pooled for each animal. At the end of the trial, daily feed samples were pooled for each dietary treatment. Feed, refusal, and faeces were dried at 65°C for 48 h, ground to pass a 1-mm sieve and sealed in bottles for chemical composition analysis.

### Rumen and Blood Sampling

Rumen samples (*ca*. 50 ml) for the analysis of rumen fermentation parameters were taken using an oesophageal tube with the first 50 ml discarded to minimise saliva contamination in a procedure described by Huo et al. (21). pH value was immediately measured using a pH metre (LICHEN pH-100 A, Shanghai Lichen Scientific Laboratory Instrument Ltd., Shanghai, China). The samples for the rumen microbial community analysis were kept in 10-ml tubes, immediately placed in liquid nitrogen, and then stored at −80°C. Rumen samples for the analysis of fermentation parameters were kept on ice in a chilly bin and transferred to the laboratory within 0.5 h. Subsamples were taken and stored in 2-ml cryogenic vials (Corning Inc., New York, USA) at −20°C until further analysis. Blood was sampled from the jugular vein and placed in a coagulation promoting tube (5 mL) with separating gel (Sanli Industrial Co., Ltd., Huizhou, China). Serum was separated by centrifuging at 1,000 × *g* for 5 min (Model TDL-80-2B; Anting Scientific Instrument Factory, Shanghai, China) and then stored at −20°C.

### Slaughter

After fasting for 24 h, lambs were weighed and slaughtered, and hot carcass weight (HCW), backfat thickness, loin eye area, and fresh organs were quantified. HCW was recorded with kidneys included. The dressing percentage was calculated as HCW divided by live body (BW) ×100. Backfat thickness was measured in the method of Kirton et al. (30). The loin eye area was estimated using the equation that eye muscle area = loin eye width and loin eye height × 0.7 (31), where the width and height were measured from the cut surface of *longissimus dorsi* muscle between the 12th and 13th ribs using a digital Vernier calliper with a precision of 0.01 mm (DL91150; Deli Group Ltd, Linbo, Zhejiang, China). Fresh organs were measured using an electronic scale with a precision of 1 g (DH-2012; Diheng Electronic Co., Ltd, Shenzhen, Guangdong, China). Organ indexes were calculated as organ weights divided by live BW.

### Laboratory Analysis

The live yeast cells in the YC products and diets were counted under a microscope after staining with alkaline methylene blue (32).

Feed samples were determined for dry matter (DM) (33), ash (34), CP [method no. 968.06; (35)], neutral detergent fibre (NDF) (36), acid detergent fibre (ADF) (36), ether extract (EE) (37), Ca (38), and P (39). Organic matter (OM) was calculated as 1,000 – ash. The NDF was assayed with a heat-stable amylase and expressed exclusive of residual ash. The ADF was consecutively determined after NDF determination and expressed exclusive of residual ash. Refusal and faeces samples were determined for DM, ash, CP, NDF and ADF, and acidified faeces samples for CP only. Urine samples were determined only for CP.

Rumen samples were determined for ammonia concentration in a modified Indigo phenol blue-spectrophotometry method (40). Short-chain fatty acids (SCFA) were identified and quantified using gas chromatography (Model GC-9A; Shimadzu Co., Japan) coupled with a flame ionisation detector (FID) and a polar capillary column (CP-WAX, 30 m × 0.53 mm × 1 μm) in a procedure described by Wang et al. (41). Microbial protein (MCP) was quantified using the spectrophotometric and HPLC methods of Zinn and Owens (42) as modified by Makkar and Becker (43) with yeast RNA as a standard.

Serum metabolites were analysed after thawing in a fridge overnight using an automatic biochemical analyser (Model 7160; Hitachi Ltd., Tokyo, Japan) with reagents from Mairui Biomedical Electronics Co., Ltd. (Shenzhen, China).

### Rumen Microbial Community Analysis

After the extraction of total genome DNA, bacterial 16S rRNA gene regions were amplified using PCR primers (341F: 5′-CCTAYGGGRBGCASCAG-3′; 806R: 5′-GGACTACNNGGGTATCTAAT-3′) as described previously (21). After sequencing libraries were generated and quality ensured, sequencing was conducted on the Illumina MiSeq platform (Illumina, Inc., San Diego, CA, USA) to generate 250 bp/300 bp paired-end reads and merged using FLASH (44). Sequence analysis was performed by the UPARSE software package using the UPARSE-OTU and UPARSE-OTU ref algorithms (45). Sequences with a similarity over 97% were treated as the same operational taxonomic unit (OTU). The classification and annotation of each OTU were performed by the RDP classifier (http://rdp.cme.msu.edu) against the Silva 123 (www.arb-silva.de) 16S rRNA database updated for ruminal bacteria (46). The relative abundances of taxa and diversity indices, including Chao, Shannon and Simpson, were estimated using the Mothur software package v.1.21.1 (47).

### Statistical Analysis

A two-way ANOVA was performed with the proportion of corn in the diet and the supplementation of YC as the experimental factors using the GenStat 19th edition statistical software (48). The multiple comparisons among treatments were performed using the Duncan method. The effect was declared as a significant difference if *P* < 0.05 and a trend if *P* < 0.10.

## Results

### Live Yeast Counts and Growth Performance

The number of live yeast cells was 2 × 10^8^ colony-forming units (cfu)/g in the YC product, and <10 × 10^3^ cfu/g in the diets supplemented either with or without YC.

Corn proportion and its interaction with YC supplementation had no significant effects on ADG and the final BW at the end of the growth performance measurement period, but YC supplementation tended to increase the final BW by 2.6 kg (*P* = 0.063) and increased ADG by 31 g/day (9.6%; *P* = 0.019) compared with CON (Table 2).

**Table 2:** Growth performance of fattening lambs fed experimental diets containing a low (LC) or high (HC) proportion of corn and supplemented with nil (CON) or yeast culture (YC) (*n* = 14 per treatment).

	**LC**		**HC**			***P*** **value**		
**Item**	**CON**	**YC**	**CON**	**YC**	**SEM**	**Corn**	**YC**	**Corn × YC**
BW^a^ d 0 (kg)	26.0	26.5	26.2	26.9	0.89	0.740	0.489	0.904
BW d 56 (kg)	44.7	46.2	43.3	47.0	1.36	0.812	0.063	0.414
ADG^b^ d 0–56 (g)	335	358	312	352	12.9	0.273	0.019	0.499

*aBW, Body weight.*

*bADG, Average daily gain*.

### Apparent Total Tract Digestibility

There were no significant interactions between the corn proportion and YC supplementation on DM and nutrient intake, digestibility, and N balance (Table 3). The supplementation of YC did not affect DM and OM intake and the digestibility of DM, OM, and CP, but resulted in an increase in NDF digestibility by 46 g/kg (*P* = 0.031) and in ADF digestibility by 71 g/kg (*P* = 0.014). The YC supplementation tended to reduce N output from urine (*P* = 0.067) and total N output (*P* = 0.045) per unit of N intake and consequently increased the proportion of retained N (*P* = 0.045). The increase in the proportion of corn in the diet decreased nutrient intake (*P* ≤ 0.004) and increased nutrient digestibility (*P* ≤ 0.016). The HC reduced daily N intake (*P* < 0.001) and daily faecal (*P* < 0.001) and urinary (*P* = 0.008) N outputs and did not change daily N retention overall. In terms of per unit of N intake, HC resulted in a decrease in faecal N output (*P* = 0.002), although there was no significant change in urinary N output and *N* retention was similar between LC and HC.

**Table 3:** Intake, total tract apparent nutrient digestibility, and nitrogen balance of fattening lambs fed experimental diets containing a low (LC) or high (HC) proportion of corn and supplemented with nil (CON) or yeast culture (YC) (*n* = 6 per treatment).

	**LC**		**HC**			***P*** **value**		
**Item**	**CON**	**YC**	**CON**	**YC**	**SEM**	**Corn**	**YC**	**Corn × YC**
**Intake (g/day)**								
Dry matter intake (DMI)	1,629	1,633	1,219	1,393	71.7	0.004	0.389	0.409
Organic matter intake (OMI)	1,531	1,579	1,176	1,375	98.1	0.010	0.223	0.453
Crude protein intake (CP intake)	271	295	197	234	12.0	<0.001	0.09	0.703
Neutral detergent fibre intake (NDF intake)	558	611	306	304	29.3	<0.001	0.414	0.380
Acid detergent fibre intake (ADF intake)	333	337	114	120	15.7	<0.001	0.767	0.967
**Digestibility (g/kg of DM)**								
Dry matter (DM)	613	606	796	787	12.3	<0.001	0.655	0.955
Organic matter (OM)	648	634	813	812	17.1	<0.001	0.666	0.692
Crude protein (CP)	713	721	771	780	11.1	0.001	0.613	0.965
Neutral detergent fibre (NDF)	221	300	414	427	18.6	<0.001	0.031	0.110
Acid detergent fibre (ADF)	133	159	142	257	22.9	0.016	0.014	0.089
***N*** **Balance**								
*N* intake (g/day)	43.4	47.2	31.5	37.4	1.93	<0.001	0.09	0.703
**Faecal** ***N*** **output**								
g/kg *N* intake	256	247	204	196	10.0	0.002	0.559	0.974
g/day	11.1	11.6	6.6	7.4	1.93	<0.001	0.425	0.856
**Urinary** ***N*** **output**								
g/kg *N* intake	440	322	402	253	48.8	0.446	0.067	0.826
g/day	18.3	15.6	11.4	9.3	1.59	0.008	0.296	0.914
**Total** ***N*** **output**								
g/kg *N* intake	696	569	606	449	46.9	0.131	0.045	0.825
g/day	29.4	27.2	18.0	16.7	1.59	<0.001	0.44	0.863
***N*** **Retention**								
g/kg *N* intake	304	431	394	551	46.9	0.131	0.045	0.825
g/day	14.0	20.0	13.5	20.8	2.17	0.967	0.043	0.832

### Rumen Fermentation Parameters

The rumen fermentation parameters (Table 4), including pH value, ammonia, MCP, total SCFA concentration, the molar proportions of acetate, propionate, and *iso*-butyrate in the total SCFA, and the ratio of acetate to propionate, were affected only by the proportion of corn in the diet (*P* ≤ 0.043), not by YC supplementation and its interaction with corn proportion. The molar proportions of butyrate, valerate, and *iso*-valerate in the total SCFA were not significantly affected by the two experimental factors.

**Table 4:** Rumen fermentation parameters of fattening lambs fed experimental diets containing a low (LC) or high (HC) proportion of corn and supplemented with nil (CON) or yeast culture (YC), sampled 3 h after morning feeding (*n* = 6 per treatment).

	**LC**		**HC**			***P-*****value**		
**Item**	**CON**	**YC**	**CON**	**YC**	**SEM**	**Corn**	**YC**	**Corn × YC**
pH	5.97	6.03	5.57	5.48	0.146	0.009	0.900	0.640
Ammonia (mM)	12.1	13.8	18.4	15.6	1.84	0.043	0.739	0.254
Microbial protein (MCP, g/L)	3.55	3.86	7.79	6.28	1.218	0.017	0.610	0.478
Short-chain fatty acids (SCFA, mM)	106.3	106.2	139.6	131.2	7.46	0.002	0.567	0.601
Acetate (mol/100 mol)	41.6	38.4	34.1	34.3	1.08	<0.001	0.201	0.147
Propionate (mol/100 mol)	41.0	43.6	51.1	52.7	1.79	<0.001	0.279	0.774
Acetate: Propionate	1.02	0.89	0.67	0.65	0.038	<0.001	0.080	0.148
Butyrate (mol/100 mol)	13.7	15.1	11.9	10.8	2.36	0.236	0.983	0.596
*iso*-Butyrate (mol/100 mol)	0.1	0.1	0.0	0.0	0.03	0.016	0.698	0.681
Valerate (mol/100 mol)	2.9	2.4	2.5	1.8	0.45	0.291	0.181	0.819
*iso*-Valerate (mol/100 mol)	0.7	0.5	0.3	0.5	0.15	0.249	0.881	0.114

### Serum Parameters

The supplementation of YC did not interact with the proportion of corn in the diet for all serum biochemical parameters assayed (Table 5). The supplementation of YC resulted in a decrease in the contents of total protein (TP; *P* = 0.002), albumin (ALB; *P* = 0.016), and globulin (GLOB; *P* = 0.017) in serum and tended to decrease the content of glucose (*P* = 0.098). The increase in the proportion of corn in the diet tended to reduce alkaline transaminase (ALT; *P* = 0.061).

**Table 5:** Serum biochemical parameters of fattening lambs fed experimental diets containing a low (LC) or high (HC) proportion of corn and supplemented with nil (CON) or yeast culture (YC) (*n* = 6 per treatment).

	**LC**		**HC**			***P-*****value**		
**Item**	**CON**	**YC**	**CON**	**YC**	**SEM**	**Corn**	**YC**	**Corn × YC**
**Protein metabolism**								
Total protein (TP, g/L)	77.0	69.2	80.2	71.0	2.25	0.218	0.002	0.747
Albumin (ALB, g/L)	31.0	30.3	32.5	29.5	0.67	0.499	0.016	0.105
Globulin (GLOB, g/L)	45.9	38.9	47.7	41.5	2.48	0.342	0.017	0.874
Albumin/Globulin	0.678	0.783	0.708	0.727	0.0442	0.753	0.173	0.355
Urea nitrogen (UREA, mmol/L)	7.92	8.07	7.56	8.18	0.647	0.822	0.578	0.726
**Energy substrates and enzymes**								
Glucose (mmol/L)	5.12	4.69	5.53	4.83	0.315	0.350	0.098	0.682
Triglyceride (TG, μmol/L)	0.286	0.243	0.214	0.200	0.0409	0.226	0.519	0.753
Total cholesterol (TC, mmol/L)	1.97	2.18	2.14	2.00	0.163	0.977	0.753	0.311
High density lipoprotein cholesterol (HDL, mmol/L)	9.28	10.10	9.16	8.32	0.707	0.214	0.990	0.278
Low density lipoprotein cholesterol (LDL, mmol/L)	0.717	0.847	0.963	0.782	0.0889	0.343	0.904	0.113
α-amylase (α-AMY, U/L)	22.5	14.9	13.8	23.0	4.91	0.888	0.935	0.109
Lipase (LIP, U/L)	39.9	44.3	38.7	53.8	7.47	0.659	0.231	0.492
**Liver function**								
Alanine transaminase (ALT, U/L)	34.2	38.4	22.3	28.3	5.54	0.061	0.385	0.870
Aspartate transaminase (AST, U/L)	182	191	177	159	26.1	0.520	0.879	0.627
Alkaline phosphatase (ALP, U/L)	394	485	490	464	59.7	0.537	0.574	0.351

### Slaughter Performance, Organ Weights and Indexes, and Digestion Tract pH Values

The proportion of corn in the diet, the supplementation of YC, and their interaction did not affect body weight at slaughter, HCW, and dressing out percentage (Table 6). The proportion of corn in the diet and its interaction with YC supplementation had no effect on the dorsal fat thickness and loin eye area, whereas YC supplementation increased dorsal fat thickness (*P* = 0.039) and tended to reduce eye muscle area (*P* = 0.062).

**Table 6:** Slaughter performance, organ weights and indexes, and digestive tract pH values of fattening lambs fed experimental diets containing a low (LC) or high (HC) proportion of corn and supplemented with nil (CON) or yeast culture (YC) (*n* = 6 per treatment).

	**LC**		**HC**			***P-*****value**		
**Item**	**CON**	**YC**	**CON**	**YC**	**SEM**	**Corn**	**YC**	**Corn × YC**
Body weight (kg)	47.3	49.3	47.8	50.5	2.38	0.719	0.338	0.890
Hot carcass (kg)	20.8	21.5	21.5	22.6	1.06	0.413	0.388	0.886
Dressing percentage (%)	43.9	43.7	45.0	44.7	0.69	0.139	0.698	0.947
Dorsal fat thickness (mm)	1.98	2.70	2.05	2.79	0.332	0.810	0.039	0.982
Eye muscle area (cm^2^)	13.2	10.7	14.1	11.1	1.39	0.654	0.062	0.849
**Organ weight (g)**
Heart	172	182	179	200	8.3	0.140	0.078	0.538
Liver	935	982	956	1 091	56.2	0.260	0.121	0.442
Spleen	68	70	74	112	9.0	0.013	0.040	0.059
Lung	540	472	439	480	43.0	0.276	0.811	0.234
Kidney	136	133	132	146	6.6	0.479	0.369	0.234
**Organ index (%)**								
Heart	0.36	0.37	0.38	0.40	0.008	0.024	0.097	0.317
Liver	1.98	1.99	2.00	2.17	0.064	0.133	0.156	0.235
Spleen	0.14	0.14	0.16	0.22	0.017	0.011	0.073	0.058
Lung	1.14	0.99	0.92	0.95	0.078	0.115	0.525	0.269
Kidney	0.29	0.27	0.28	0.29	0.010	0.480	0.817	0.114
**pH value**								
Rumen	6.70	6.65	6.67	6.65	0.111	0.894	0.755	0.929
Reticulum	6.92	6.78	6.65	6.68	0.062	0.008	0.480	0.200
Omasum	5.60	5.91	5.96	5.57	0.206	0.949	0.861	0.100
Abomasum	3.53	3.59	4.27	3.84	0.383	0.216	0.639	0.528
Cecum	6.57	6.46	6.06	6.31	0.093	0.002	0.478	0.067

The supplementation of YC had no effect on spleen weight and the proportion of spleen in the whole body at LC; however, at HC, the effects were significant (*P* < 0.05). The heart tended to be heavier, and the proportion of heart in the whole body tended to be higher after supplementation with YC (*P* < 0.010). The pH values of the contents of the reticulum and cecum were lower for HC than for LC (*P* < 0.05), but the supplementation of YC did not affect digestion tract pH values.

### Rumen Microbial Communities

The number of OTUs was not affected by either the supplementation of YC or the proportion of corn in the diet (Table 7) and averaged 947. The community richness index Chao was not significantly affected by the proportion of corn in the diet, the supplementation of YC, and their interaction. The Shannon index is one of the indexes for estimating microbial diversity in samples. The index was affected by both experimental factors. The index dropped from 4.25 to 3.98 when the proportion of corn in the diet increased from 350 to 600 g/kg (*P* < 0.001) and the supplementation of YC also reduced the index from 4.19 to 4.04 (*P* = 0.036).

**Table 7:** The rumen bacterial diversity of fattening lambs fed experimental diets containing a low (LC) or high (HC) proportion of corn and supplemented with nil (CON) or yeast culture (YC) (*n* = 12 per treatment).

	**LC**		**HC**			***P-*****value**		
**Item**	**CON**	**YC**	**CON**	**YC**	**SEM**	**Corn**	**YC**	**Corn × YC**
Reads	47,037	41,769	42,908	47,530	2,374.0	0.733	0.892	0.043
OTU^a^	989	970	947	898	34.0	0.100	0.330	0.662
Coverage	0.993	0.992	0.992	0.994	0.0005	0.349	0.726	0.014
Chao	1,340	1,336	1,289	1,234	45.5	0.100	0.530	0.578
Shannon	4.30	4.20	4.08	3.87	0.069	<0.001	0.036	0.463
Simpson	0.0495	0.0459	0.0487	0.0602	0.00424	0.117	0.358	0.081

*^a^OTU, operational taxonomic unit*.

*Bacteroidetes, Firmicutes*, and *Proteobacteria* were the three most abundant phyla, accounting for 55, 29, and 14% of the total bacterial sequences analysed, respectively, and summing up to 98% (Table 8). The proportion of corn in the diet and the supplementation of YC did not affect the abundance of these phyla with an exception that HC tended to increase the abundance of *Proteobacteria* compared with LC (*P* = 0.090). The fourth and fifth abundant phyla were *Fibrobacteres* and *Spirochaetes*, and the remainder of the phyla identified were <1% in any one of the four treatment groups. Increases in the proportion of corn in the diet decreased the abundance of *Fibrobacteres* (*P* = 0.017) and *Spirochaetes* (*P* = 0.005) from 1.3 to 0.4% and from 0.7 to 0.1%, respectively. The supplementation of YC or increasing corn proportion increased the abundance of *Cyanobacteria* (*P* ≤ 0.030). The interaction between corn proportion and YC supplementation affected the abundance of *Tenericutes*. At LC, YC supplementation reduced the abundance from 0.2 to 0.1% (*P* < 0.01). At HC, *Tenericutes* disappeared from the rumen (*P* = 0.030). The supplementation of YC resulted in the disappearance of *Patescibacteria* from the rumen (*P* = 0.022).

**Table 8:** Ruminal bacterial phyla (being higher than 0.1% of the total bacteria at least in one of the four treatments) in fattening lambs fed experimental diets containing a low (LC) or high (HC) proportion of corn and supplemented with nil (CON) or yeast culture (YC) (*n* = 12 per treatment), expressed as a percentage of the total.

	**LC**		**HC**			***P-*****value**		
**Item**	**CON**	**YC**	**CON**	**YC**	**SEM**	**Corn**	**YC**	**Corn × YC**
Bacteroidetes	54.4	56.1	56.6	52.8	2.88	0.845	0.722	0.341
Firmicutes	31.2	28.3	27.8	26.6	3.23	0.433	0.516	0.798
Proteobacteria	10.5	13.1	14.5	18.8	2.82	0.090	0.229	0.770
Fibrobacteres	1.3	1.2	0.1	0.7	0.34	0.017	0.402	0.357
Spirochaetes	1.0	0.4	0.0	0.1	0.20	0.005	0.157	0.101
Actinobacteria	0.4	0.2	0.3	0.2	0.09	0.395	0.270	0.621
Cyanobacteria	0.1	0.3	0.3	0.5	0.08	0.006	0.030	0.789
Tenericutes	0.2	0.1	0.0	0.0	0.03	<0.001	0.030	0.033
Synergistetes	0.1	0.1	0.1	0.2	0.08	0.678	0.649	0.238
Patescibacteria	0.1	0.0	0.1	0.0	0.04	0.775	0.022	0.437
Unclassified	0.6	0.0	0.1	0.0	0.23	0.218	0.166	0.233
Elusimicrobia	0.0	0.2	0.0	0.0	0.05	0.067	0.116	0.147

The interaction between the proportion of corn in the diet and the supplementation of YC affected the abundance of the *Rikenellaceae* RC9 gut group (*P* = 0.023), *Prevotellaceae*_Unclassified (*P* = 0.026), *Alloprevotella* (*P* = 0.028), *Oribacterium* (*P* = 0.037), and *Megasphaera* (*P* = 0.065) and tended to affect the abundance of *Prevotellaceae* UCG-001 (*P* = 0.092) but had no effect on the abundance of other genera listed in Table 9 and Supplementary Table 1. At LC, the supplementation of YC decreased the abundance of the *Rikenellaceae* RC9 gut group, *Alloprevotella*, and *Prevotellaceae* UCG-001 (*P* < 0.05) and increased the abundance of *Prevotellaceae*_Unclassified (*P* < 0.05). At HC, the supplementation of YC did not result in significant differences for these genera.

**Table 9:** The relative abundance of ruminal bacterial genera (being higher than 0.5% of the total bacteria at least in one of the four treatments and statistically different among treatments) in fattening lambs fed experimental diets containing a low (LC) or high (HC) proportion of corn and supplemented with nil (CON) or yeast culture (YC) (*n* = 12 per treatment), expressed as a percentage of the total.

	**LC**		**HC**			***P*** **value**		
**Item**	**CON**	**YC**	**CON**	**YC**	**SEM**	**Corn**	**YC**	**Corn × YC**
*Rikenellaceae* RC9 gut group	2.43	0.86	0.79	1.31	0.44	0.187	0.240	0.023
*Prevotellaceae*_Unclassified	0.43	0.75	0.84	0.73	0.09	0.037	0.260	0.026
*Alloprevotella*	0.56	0.06	0.00	0.02	0.11	0.012	0.042	0.028
*Oribacterium*	1.04	0.67	0.35	1.02	0.24	0.474	0.531	0.037
*Megasphaera*	0.59	1.16	0.49	0.17	0.24	0.026	0.597	0.065
*Prevotellaceae* UCG-001	2.10	0.85	0.21	0.18	0.36	<0.001	0.080	0.092
[Eubacterium] *coprostanoligenes* group	0.69	0.29	0.36	0.18	0.12	0.074	0.021	0.379
*Gastranaerophilales*_norank	0.12	0.27	0.32	0.51	0.08	0.010	0.035	0.800
*Ruminococcaceae* NK4A214 group	0.58	0.29	0.20	0.17	0.09	0.009	0.096	0.171
*Ruminococcus* 1	1.69	0.99	0.23	0.20	0.22	<0.001	0.101	0.135
*Christensenellaceae* R-7 group	0.92	0.31	0.08	0.03	0.21	0.010	0.124	0.187
*Mitsuokella*	0.28	0.54	0.19	0.21	0.09	0.021	0.128	0.180
*Treponema* 2	0.98	0.33	0.03	0.08	0.20	0.005	0.145	0.100
*Fibrobacter*	1.27	1.24	0.12	0.72	0.34	0.017	0.402	0.357
*F082*_norank	1.48	1.15	0.43	0.50	0.25	0.001	0.612	0.422
*Lachnospira*	0.65	0.44	0.09	0.20	0.11	<0.001	0.632	0.157
[Eubacterium] *ruminantium* group	0.89	1.04	0.07	0.11	0.24	<0.001	0.686	0.828
*Anaerobiospirillum*	0.27	0.10	0.48	0.60	0.16	0.033	0.872	0.363

Increases in the corn proportion resulted in decreases in the abundance of the [Eubacterium] *coprostanoligenes* group (*P* = 0.074), *Ruminococcaceae* NK4A214 group (*P* = 0.009), *Ruminococcus* 1 (*P* < 0.001), *Christensenellaceae* R-7 group (*P* = 0.010), *Mitsuokella* (*P* = 0.021), *Treponema* 2 (*P* = 0.005), *Fibrobacter* (*P* = 0.017), *F082_norank* (*P* = 0.001), *Lachnospira* (*P* < 0.001), and [Eubacterium] *ruminantium* group (*P* < 0.001), and increases in *Gastranaerophilales*_norank (*P* = 0.010) and *Anaerobiospirillum* (*P* = 0.033). Among these genera, the [Eubacterium] *coprostanoligenes* group (*P* = 0.021) and *Ruminococcaceae* NK4A214 group (*P* = 0.096) decreased, and *Gastranaerophilales*_norank (*P* = 0.035) increased with the supplementation of YC, cellulolytic bacteria *Fibrobacter, Ruminococcus* 1, and *Butyrivibrio* 2, and other bacteria were not significantly affected. Lactate-producing bacteria *Lactobacillus, Streptococcus*, and *Bifidobacterium* were < 0.1% in total bacteria and thus not listed in Supplementary Table 1. Lactate-consuming bacteria *Megasphaera* and *Selenomonas 3* were < 1.2% and 0.5% in any one of the four treatment groups and not affected by YC supplementation.

## Discussion

The YC product used in this study contained 2 × 10^8^ cfu/g live yeast cells and was supplemented at 5 g/kg feed, which was expected to have 1 × 10^6^ cfu/g live cells in the feed if all live yeast cells survived. However, the results from the microscopical evaluation showed that the number of live yeast cells was <10 × 10^3^ cfu/g with a survival rate of <1%, which was the same as the CON, indicating that live yeast in the YC product was inactivated during the process of pelleting. Yeast is sensitive to high temperatures (49) and would not survive during pelleting without being protected using special techniques such as agglomeration (50, 51) or micro-encapsulation (52). The loss of yeast viability during pelleting in the present study suggests that the effects of the YC supplementation, if they occur, are from dead yeast, the metabolites of yeast fermentation, and culture media rather than from live yeast. Thus, we can only compare our results with other YC studies in which live yeast does not play a significant role.

In the present study, 31.1 g/day more ADG resulted from the supplementation of YC during the 56-day animal growth performance measurement period. This result is consistent with the finding of more N retained in lambs supplemented with YC and indicated beneficial biological effects of YC on animal growth. The increase of 9.6% in ADG with YC supplementation is consistent with the report by Ghoneem and Mahmoud (53) who provided each lamb 5 g of inactivated yeast per day, which is equivalent to 4.7 g/kg feed and similar to the dose we had in this study but is contrary to the findings of Soliman et al. (54). However, in the study by Soliman et al. (54), each lamb was given 10 g of prebiotics per day containing on a per-kilogramme basis, 100 g of inactive yeast product (50% mannan + 50% β-glucan), 2 g of MnSO_4_.H_2_O, 0.12 g of vitamin A, 0.13 g of vitamin E, and 500 g of bentonite, and the feed intake was 1,197 g per day. The real dose of yeast product was only 0.84 g/kg feed, which was largely lower than the dose of 5 g/kg feed in our study. The low dose might be a reason for the lack of improvement in animal growth performance in their study. Our results are in agreement with the studies on other ruminants; for example, Lei et al. (55) fed 2 g/kg yeast cell walls to beef cattle, resulting in an increase of 12.8% ADG, and Peng et al. (56) found an increase of 9.5% ADG when beef cattle were supplemented with yeast cell walls at 20 g/day/head.

Animal growth performance reflects the feeding value of a feed, which is the function of feed intake and feed nutritional value. Feed intake depends on the physical capacity of the digestive system, the physical and chemical properties of the diet, and the digestion and utilisation of nutrients in ruminant animals (57). The supplementation of YC does not change the dietary chemical composition and physical characteristics. Thus, we would not expect an increase in feed intake with YC supplementation. This is confirmed with our result that feed intake was similar between the presence and absence of YC supplementation in the diet observed during the digestibility measurement period, and this result is consistent with the results of Ghoneem and Mahmoud (53) and Soliman et al. (54), suggesting that the improved growth performance with the supplementation of YC may not be related to feed intake.

The fibre digestibility increased with the supplementation of YC, although DM, OM, and CP digestibility did not change in this study. These findings are in agreement with a summary by Bakory (58) who reviewed a range of studies with YC supplemented to ruminants and with a study where beef cattle were fed a diet containing 2 g of yeast cell walls per kg of diet (55). In a study on the supplementation of YC to growing lambs, the digestibility of DM, OM, CP, NDF, and ADF all improved and the extent of increase in fibre digestibility was much higher than our study (59). Our results are also consistent with a dairy cow study where the supplementation of killed yeast tended to increase fibre digestibility, although lactation performance was not improved (60). Although some discrepancies exist among these studies, the common finding is an increase in fibre digestibility with the supplementation of YC. The abundance of fibre-degrading bacteria would be expected to increase for the increased fibre digestibility as found by Abou Elenin et al. (59). It is proposed that metabolites in YC stimulate cellulolytic bacteria (61). However, we did not find an increased abundance of fibre-degrading bacteria in the rumen with the supplementation of YC. This agrees with studies by Dawson et al. (62) and Jiang et al. (60) who found that inactive yeast was unable to stimulate the growth of cellulolytic bacteria. There would be other explanations for increased fibre digestibility with unchanged cellulolytic bacteria. One possibility is that YC may stimulate anaerobic fungi colonising fibre to make the fibre more accessible to fibre-degrading bacteria rather than having a direct effect on the proliferation of these bacteria (58). Live yeast can stimulate the growth of fibre-degrading fungus *Neocallimastix frontalis* (63). Inactive yeast might have the same effect, but this needs experimental evidence to support.

The contents of TP, ALB, and GLOB in serum decreased with the supplementation of YC in this study. These findings were contradictory to the results obtained from lambs (10, 59, 64) and from dairy cows (7). Even the results among these studies were inconsistent. The heterogeneity in response was claimed to be a result of the delivery method of YC (top-dressing vs. mixing with the TMR) (7). The major difference between our study and these studies was that pelleting was conducted for the YC-supplemented TMR and resulted in the loss of live yeast activity, which might be a reason for the difference. Blood urea nitrogen is also related to protein metabolism and is an indirect indicator of dietary protein composition and/or utilisation. The supplementation of YC did not change blood urine nitrogen concentration in our study and in the literature (7, 64), suggesting nitrogen status was unchanged with the supplementation of YC. Energy-related parameters such as serum glucose, triglyceride, and total cholesterol were not affected by YC supplementation. These results are consistent with studies on lambs (64) and on dairy cows (7). Parameters related to liver function such as ALT, AST, and ALP were also not changed with YC in this study and in the study by Abou Elenin et al. (59), suggesting that YC has no adverse or beneficial effects on liver metabolism.

Although HCW was 3.7–5.1% higher when YC was supplemented to both the LC and HC diets than CON, the differences were not statistically significant. In most YC supplement studies where even yeast is active, slaughter performance does not change (5, 65). An increased ADG, as occurred in this study, does not necessarily translate to improved slaughter performance (66, 67). Another reason is that in most slaughter trials, six to eight lambs are applied to detect treatment differences, which do not have enough power to detect a moderate response. With our own HCW data in this study, a power analysis indicated that seven lambs are needed to detect a 20% difference in HCW, 23 lambs for a 10% difference, and 87 lambs for a 5% difference when power is set at 80% and the significance level is set at 5%.

It is well-known that the ruminal microbial community is associated with animal performance, health, and immunity (68). Both the supplementation of YC and the proportion of corn in the diet altered ruminal microbial community in lambs in our study. However, the most abundant three phyla *Bacteroidetes, Firmicutes*, and *Proteobacteria* were predominant in the rumen, being over 98% of the total bacteria, and were not affected by the two experimental treatments, which is consistent with the findings by Li et al. (69) and supports the existence of a core rumen microbiota claimed by Henderson et al. (70). The relative abundance of *Prevotella* was around half of the total bacteria, reflecting the importance of this microbe in the rumen for its roles in carbohydrate utilisation, nitrogen metabolism, and fibre degradation (69).

Our results showed that the relative abundances of the *[Eubacterium] coprostanoligenes* group and *Ruminococcaceae* NK4A214 group decreased with the supplementation of YC. These two genera were found to have higher abundances in the high-yielding dairy cows than in the low-yielding cows (71). The *[Eubacterium] coprostanoligenes* group was reported to have a negative correlation with milk fat content (72), suggesting that lipid metabolism might be modulated via the bacteria, and this could be a reason for the increased dorsal fat thickness with the supplementation of YC in our study. *Ruminococcaceae* could have a critical role in biohydrogenation pathways in the rumen (73). Knowledge of the biological functions of these two genera in the rumen reported in the literature so far is too little to explain the altered abundance of the genera in lambs with the supplementation of YC and thus warrants further studies. In contrast to the above genera, *Gastranaerophilales*_norank increased with the supplementation of YC. The relative abundance of the order *Gastranaerophilales* in the rumen had a negative correlation with milk yield in dairy cows (74), and lambs with low feed efficiency had a higher relative abundance of *Gastranaerophilales* than those with high feed efficiency (75). These results are contradictory to our results that found that *Gastranaerophilales* was associated with higher ADG with the supplementation of YC. This discrepancy is difficult to explain based on the knowledge we have from the literature.

Lambs in the HC diet had lower relative abundances of *Ruminococcus* 1, *Christensenellaceae* R-7 group, *Mitsuokella, Treponema 2, Fibrobacter, F082*_norank, *Lachnospira*, and *[Eubacterium] ruminantium* group. Some of these microbes are fibre-degrading bacteria, such as *Ruminococcus* 1, *Fibrobacter* (76), and *Treponema* (77). It is not surprising that fibre-degrading bacteria decline under a high-grain diet. There were several genera, such as the *Rikenellaceae* RC9 gut group and *Prevotellaceae* UCG-001, with their relative abundances affected by the interaction between the proportion of corn in the diet and YC supplementation, which is a challenge for the interpretation of these data.

We did not find reportable amounts of typical lactate-producing bacteria *Lactobacillus, Streptococcus*, and *Bifidobacterium*, and lactate-consuming bacteria *Megasphaera* and *Selenomonas 3* (14) were in the minimum amount for reporting. No rumen pH values were significantly lower than 5.5. These results suggested that even lambs fed the HC diet did not suffer from acidosis and the role of YC in the stabilisation of rumen pH did not appear.

## Conclusions

In conclusion, live yeast cells in the YC product could not survive during TMR pelleting. The supplementation of YC to pelleted TMR at a dose of 5 g/kg improved male lamb growth performance regardless of the proportion of corn in the diet. The improvement might not be attributed to feed intake but could be related to increased fibre digestibility, although the relative abundance of fibre-degrading bacteria in the rumen did not increase. Dorsal fat thickness increased with the supplementation of YC, and eye muscle area tended to decrease. The supplementation of YC altered rumen bacterial community although the core rumen microbiota remained unchanged. Although pelleting inactivates live yeast, bioactive substances in the YC product supplemented to pelleted TMR still improved lamb growth performance and thus YC as a feed additive can be feasibly included in pelleted TMR for sheep.

## Data Availability Statement

Sequencing data are available in the NCBI Sequence Read Archive (http://www.ncbi.nlm.nih.gov/bioproject/694322), as project PRJNA694322, submission ID SUB8925435. All the rest of the relevant data are within the paper and its supporting information file.

## Ethics Statement

The animal study was reviewed and approved by the Animal Ethics and Welfare Committee of Jilin Agricultural Science and Technology University (Approval number 2018005).

## Author Contributions

XS, PY, HW, and KK conceived and planned the study. XS acquired funding, supervised all research, analysed and interpreted data, prepared the tables, prepared the figures, and wrote the manuscript. BS, TW, WY, MW, BL, YH, QH, CL, WT, RL, JL, CW, and XS collected data. XS and JB revised the manuscript. All authors contributed to the article and approved the submitted version.

## Conflict of Interest

PY and WY were employed by Portal Agri-Industries Co., Ltd. HW and KK were employed by Angel Yeast Co., Ltd. The remaining authors declare that the research was conducted in the absence of any commercial or financial relationships that could be construed as a potential conflict of interest. The funders of the study were not involved in the study design, the collection, analysis, and interpretation of data, the writing of this article or the decision to submit it for publication.
