# Supplementary material for: Dietary Supplementation of Yeast Culture Into Pelleted Total Mixed Rations Improves the Growth Performance of Fattening Lambs
Source: Front Vet Sci. 2021 May 12;8:657816. doi: 10.3389/fvets.2021.657816 (PMC8149762; doi:10.3389/fvets.2021.657816)
Supplement: Supplementary file 1 [file Table_1.DOCX]

| **Supplementary Table 1 \|** Ruminal bacterial genera with at least one of the four treatments higher than 0.1% in total bacteria in fattening lambs fed experimental diets containing a low (LC) or high (HC) proportion of corn and supplemented with nil (CON) or yeast culture (YC) (n=12 per treatment), expressed as a percentage of the total. | | | | | | | | | |
| --- | --- | --- | --- | --- | --- | --- | --- | --- | --- |
|  |  |  |  |  |  |  |  |  |  |
|  | **LC** | |  | **HC** | |  | ***P* value** | | |
| **Item** | **CON** | **YC** |  | **CON** | **YC** | **SEM** | **Corn** | **YC** | **Corn × YC** |
| *Prevotella* 7 | 27.8 | 48.5 |  | 52.1 | 46.5 | 4.17 | 0.011 | 0.078 | 0.003 |
| *Succinivibrionaceae* UCG-001 | 8.0 | 12.7 |  | 13.7 | 15.5 | 2.77 | 0.130 | 0.252 | 0.604 |
| *Veillonellaceae*_uncultured | 5.2 | 6.0 |  | 8.0 | 5.6 | 1.73 | 0.499 | 0.663 | 0.366 |
| *Prevotella* 1 | 15.8 | 1.1 |  | 0.2 | 0.3 | 2.59 | 0.003 | 0.007 | 0.007 |
| *Dialister* | 2.5 | 3.2 |  | 4.5 | 3.0 | 0.62 | 0.156 | 0.513 | 0.084 |
| *Syntrophococcus* | 1.4 | 4.1 |  | 3.6 | 4.6 | 1.11 | 0.221 | 0.109 | 0.442 |
| *Lachnospiraceae* NK3A20 group | 1.5 | 2.4 |  | 1.0 | 2.2 | 0.75 | 0.666 | 0.147 | 0.848 |
| *Ruminococcaceae* UCG-014 | 1.4 | 1.6 |  | 1.8 | 1.6 | 0.49 | 0.753 | 0.945 | 0.727 |
| *Rikenellaceae* RC9 gut group | 2.4 | 0.9 |  | 0.8 | 1.3 | 0.44 | 0.187 | 0.240 | 0.023 |
| *Shuttleworthia* | 0.2 | 0.5 |  | 3.1 | 2.6 | 1.54 | 0.111 | 0.942 | 0.806 |
| *Muribaculaceae*_norank | 1.3 | 1.1 |  | 1.1 | 2.1 | 0.57 | 0.517 | 0.464 | 0.314 |
| *Succinivibrio* | 1.7 | 0.0 |  | 0.0 | 0.1 | 0.62 | 0.188 | 0.193 | 0.170 |
| *F082*_norank | 1.5 | 1.2 |  | 0.4 | 0.5 | 0.25 | 0.001 | 0.612 | 0.422 |
| *Prevotellaceae* UCG-001 | 2.1 | 0.9 |  | 0.2 | 0.2 | 0.36 | <0.001 | 0.080 | 0.092 |
| *Fibrobacter* | 1.3 | 1.2 |  | 0.1 | 0.7 | 0.34 | 0.017 | 0.402 | 0.357 |
| *Succiniclasticum* | 0.8 | 0.8 |  | 0.5 | 0.8 | 0.35 | 0.648 | 0.649 | 0.682 |
| Prevotellaceae_Unclassified | 0.4 | 0.7 |  | 0.8 | 0.7 | 0.09 | 0.037 | 0.260 | 0.026 |
| *Ruminococcus* 1 | 1.7 | 1.0 |  | 0.2 | 0.2 | 0.22 | <0.001 | 0.101 | 0.135 |
| *Oribacterium* | 1.0 | 0.7 |  | 0.3 | 1.0 | 0.24 | 0.474 | 0.531 | 0.037 |
| *Megasphaera* | 0.6 | 1.2 |  | 0.5 | 0.2 | 0.24 | 0.026 | 0.597 | 0.065 |
| *Gastranaerophilales*_norank | 0.1 | 0.3 |  | 0.3 | 0.5 | 0.08 | 0.010 | 0.035 | 0.800 |
| *Prevotella* 9 | 0.4 | 0.3 |  | 0.4 | 0.5 | 0.24 | 0.753 | 0.986 | 0.527 |
| *Acidaminococcus* | 0.3 | 0.5 |  | 0.6 | 0.5 | 0.11 | 0.223 | 0.794 | 0.252 |
| *Succinatimonas* | 0.1 | 0.0 |  | 0.0 | 2.0 | 0.97 | 0.342 | 0.338 | 0.309 |
| [Eubacterium] *ruminantium* group | 0.9 | 1.0 |  | 0.1 | 0.1 | 0.24 | <0.001 | 0.686 | 0.828 |
| *Selenomonas* 3 | 0.5 | 0.3 |  | 0.2 | 0.4 | 0.15 | 0.518 | 0.927 | 0.101 |
| *Treponema* 2 | 1.0 | 0.3 |  | 0.0 | 0.1 | 0.20 | 0.005 | 0.145 | 0.100 |
| *Roseburia* | 0.2 | 0.1 |  | 0.6 | 0.7 | 0.39 | 0.203 | 0.963 | 0.738 |
| [Eubacterium] *coprostanoligenes* group | 0.7 | 0.3 |  | 0.4 | 0.2 | 0.12 | 0.074 | 0.021 | 0.379 |
| *Lachnospira* | 0.6 | 0.4 |  | 0.1 | 0.2 | 0.11 | <0.001 | 0.632 | 0.157 |
| *Anaerobiospirillum* | 0.3 | 0.1 |  | 0.5 | 0.6 | 0.16 | 0.033 | 0.872 | 0.363 |
| *Christensenellaceae* R-7 group | 0.9 | 0.3 |  | 0.1 | 0.0 | 0.21 | 0.010 | 0.124 | 0.187 |
| *Ruminococcaceae* NK4A214 group | 0.6 | 0.3 |  | 0.2 | 0.2 | 0.09 | 0.009 | 0.096 | 0.171 |
| *Erysipelotrichaceae* UCG-002 | 0.3 | 0.4 |  | 0.2 | 0.3 | 0.15 | 0.487 | 0.450 | 0.791 |
| *Mitsuokella* | 0.3 | 0.5 |  | 0.2 | 0.2 | 0.09 | 0.021 | 0.128 | 0.180 |
| *Bacteroidales_*norank | 0.3 | 0.3 |  | 0.1 | 0.1 | 0.05 | 0.001 | 0.880 | 0.908 |
| *Selenomonas* | 0.5 | 0.1 |  | 0.1 | 0.0 | 0.21 | 0.185 | 0.299 | 0.448 |
| *Olsenella* | 0.3 | 0.2 |  | 0.2 | 0.2 | 0.08 | 0.565 | 0.325 | 0.623 |
| *Lachnospiraceae* ND3007 group | 1.0 | 0.0 |  | 0.0 | 0.0 | 0.44 | 0.244 | 0.270 | 0.261 |
| *Ruminococcaceae* UCG-002 | 0.2 | 0.0 |  | 0.1 | 0.1 | 0.05 | 0.153 | 0.078 | 0.064 |
| *Desulfovibrio* | 0.2 | 0.1 |  | 0.2 | 0.3 | 0.08 | 0.104 | 0.612 | 0.192 |
| *Ruminococcaceae* UCG-005 | 0.3 | 0.2 |  | 0.3 | 0.1 | 0.10 | 0.519 | 0.188 | 0.685 |
| [Ruminococcus] *gauvreauii* group | 0.4 | 0.2 |  | 0.1 | 0.1 | 0.10 | 0.032 | 0.297 | 0.121 |
| *Prevotellaceae*_uncultured | 0.1 | 0.2 |  | 0.1 | 0.2 | 0.05 | 0.701 | 0.063 | 0.921 |
| [Eubacterium] *nodatum* group | 0.3 | 0.1 |  | 0.2 | 0.1 | 0.05 | 0.260 | 0.041 | 0.346 |
| *Selenomonas* 1 | 0.9 | 0.0 |  | 0.0 | 0.0 | 0.32 | 0.178 | 0.178 | 0.178 |
| *Alloprevotella* | 0.6 | 0.1 |  | 0.0 | 0.0 | 0.11 | 0.012 | 0.042 | 0.028 |
| *Unclassified* | 0.6 | 0.0 |  | 0.1 | 0.0 | 0.23 | 0.218 | 0.166 | 0.233 |
| *Prevotellaceae* UCG-003 | 0.5 | 0.0 |  | 0.0 | 0.0 | 0.11 | 0.042 | 0.051 | 0.049 |
| *Bacteroidales* RF16 group_norank | 0.3 | 0.1 |  | 0.0 | 0.1 | 0.09 | 0.151 | 0.504 | 0.072 |
| *Lachnoclostridium* 1 | 0.2 | 0.1 |  | 0.1 | 0.3 | 0.06 | 0.837 | 0.241 | 0.024 |
| *Prevotella* | 0.1 | 0.2 |  | 0.2 | 0.1 | 0.02 | 0.267 | 0.503 | <0.001 |
| *Erysipelotrichaceae* UCG-004 | 0.4 | 0.1 |  | 0.0 | 0.1 | 0.13 | 0.133 | 0.237 | 0.152 |
| *Clostridiales vadinBB60* group_norank | 0.2 | 0.2 |  | 0.1 | 0.1 | 0.04 | 0.039 | 0.585 | 0.905 |
| *Ruminococcus* 2 | 0.6 | 0.0 |  | 0.0 | 0.0 | 0.21 | 0.151 | 0.168 | 0.162 |
| *Anaerovibrio* | 0.3 | 0.0 |  | 0.0 | 0.0 | 0.15 | 0.272 | 0.271 | 0.270 |
| *Prevotellaceae* UCG-004 | 0.2 | 0.2 |  | 0.1 | 0.1 | 0.03 | 0.006 | 0.843 | 0.654 |
| *Lachnospiraceae* UCG-008 | 0.2 | 0.1 |  | 0.0 | 0.0 | 0.03 | <0.001 | 0.080 | 0.131 |
| *Ruminococcaceae* UCG-010 | 0.2 | 0.1 |  | 0.0 | 0.0 | 0.04 | 0.003 | 0.163 | 0.231 |
| *Acetitomaculum* | 0.1 | 0.1 |  | 0.1 | 0.1 | 0.04 | 0.439 | 0.181 | 0.908 |
| *Erysipelotrichaceae* UCG-007 | 0.0 | 0.0 |  | 0.1 | 0.2 | 0.05 | 0.043 | 0.175 | 0.189 |
| *Saccharofermentans* | 0.4 | 0.0 |  | 0.0 | 0.0 | 0.10 | 0.036 | 0.067 | 0.067 |
| *Pyramidobacter* | 0.1 | 0.0 |  | 0.0 | 0.2 | 0.08 | 0.729 | 0.573 | 0.201 |
| *Bacteroidales* BS11 gut group_norank | 0.3 | 0.1 |  | 0.0 | 0.0 | 0.05 | 0.005 | 0.067 | 0.032 |
| *Catonella* | 0.1 | 0.1 |  | 0.2 | 0.1 | 0.09 | 0.546 | 0.451 | 0.714 |
| *Veillonellaceae* UCG-001 | 0.2 | 0.0 |  | 0.0 | 0.0 | 0.05 | 0.092 | 0.092 | 0.087 |
| *p-251-o5_*norank | 0.1 | 0.3 |  | 0.0 | 0.0 | 0.11 | 0.114 | 0.305 | 0.317 |
| *Moryella* | 0.2 | 0.0 |  | 0.0 | 0.0 | 0.07 | 0.124 | 0.218 | 0.104 |
| *Sharpea* | 0.1 | 0.1 |  | 0.0 | 0.0 | 0.04 | 0.028 | 0.342 | 0.192 |
| *Mollicutes* RF39_norank | 0.2 | 0.1 |  | 0.0 | 0.0 | 0.03 | <0.001 | 0.030 | 0.034 |
| *Ruminococcaceae* UCG-013 | 0.1 | 0.1 |  | 0.0 | 0.0 | 0.03 | 0.012 | 0.869 | 0.978 |
| *Lachnospiraceae_*uncultured | 0.2 | 0.0 |  | 0.0 | 0.0 | 0.04 | 0.086 | 0.182 | 0.093 |
| *Elusimicrobium* | 0.0 | 0.2 |  | 0.0 | 0.0 | 0.05 | 0.066 | 0.117 | 0.145 |
| *Defluviitaleaceae* UCG-011 | 0.1 | 0.1 |  | 0.0 | 0.0 | 0.03 | 0.019 | 0.514 | 0.206 |
| *Ruminiclostridium* 6 | 0.2 | 0.0 |  | 0.0 | 0.0 | 0.07 | 0.178 | 0.220 | 0.220 |
| *Ruminococcaceae* UCG-004 | 0.1 | 0.0 |  | 0.0 | 0.0 | 0.02 | 0.090 | 0.125 | 0.125 |
| *Saccharimonadales_*norank | 0.0 | 0.0 |  | 0.1 | 0.0 | 0.03 | 0.076 | 0.195 | 0.284 |
| *Halomonas* | 0.0 | 0.0 |  | 0.0 | 0.2 | 0.10 | 0.307 | 0.339 | 0.339 |
| *Butyrivibrio* 2 | 0.1 | 0.0 |  | 0.0 | 0.0 | 0.03 | 0.046 | 0.175 | 0.098 |
| *Candidatus Saccharimonas* | 0.1 | 0.0 |  | 0.0 | 0.0 | 0.03 | 0.042 | 0.051 | 0.057 |
| *Family XIII AD3011* group | 0.1 | 0.0 |  | 0.0 | 0.0 | 0.03 | 0.051 | 0.051 | 0.085 |
